# Bone regeneration in calvarial defects in a rat model by implantation of human bone marrow-derived mesenchymal stromal cell spheroids

**DOI:** 10.1007/s10856-015-5591-3

**Published:** 2015-10-08

**Authors:** Hideyuki Suenaga, Katsuko S. Furukawa, Yukako Suzuki, Tsuyoshi Takato, Takashi Ushida

**Affiliations:** Department of Oral-Maxillofacial Surgery, Dentistry and Orthodontics, The University of Tokyo Hospital, 7-3-1 Hongo, Bunkyo-ku, Tokyo, 113-8655 Japan; Biomedical Engineering Laboratory, Department of Bioengineering and Mechanical Engineering, Graduate School of Engineering, The University of Tokyo, Tokyo, Japan; Center for Disease Biology and Integrative Medicine, Graduate School of Medicine, The University of Tokyo, Tokyo, Japan; Division of Tissue Engineering, The University of Tokyo Hospital, Tokyo, Japan

## Abstract

Mesenchymal stem cell (MSC) condensation contributes to membrane ossification by enhancing their osteodifferentiation. We investigated bone regeneration in rats using the human bone marrow-derived MSC-spheroids prepared by rotation culture, without synthetic or exogenous biomaterials. Bilateral calvarial defects (8 mm) were created in nude male rats; the left-sided defects were implanted with MSC-spheroids, β-tricalcium phosphate (β-TCP) granules, or β-TCP granules + MSC-spheroids, while the right-sided defects served as internal controls. Micro-computed tomography and immunohistochemical staining for osteocalcin/osteopontin indicated formation of new, full-thickness bones at the implantation sites, but not at the control sites in the MSC-spheroid group. Raman spectroscopy revealed similarity in the spectral properties of the repaired bone and native calvarial bone. Mechanical performance of the bones in the MSC-implanted group was good (50 and 60 % those of native bones, respectively). All tests showed poor bone regeneration in the β-TCP and β-TCP + MSC-spheroid groups. Thus, significant bone regeneration was achieved with MSC-spheroid implantation into bone defects, justifying further investigation.

## Introduction

Congenital and acquired cranial bone defects occur widely worldwide and are therapeutically challenging [1, 2]. Current treatment approaches include autografts, allografts, and scaffolds made of osteoconductive materials. Drawbacks of autograft transplantation are limited supply, donor-site pain, and overall morbidity, while those of allograft transplantation are host rejection, infection, disease transmission, and inflammation [3, 4]. The osteoconductive biomaterials hydroxyapatite and beta-tricalcium phosphate (β-TCP) used for synthetic ceramic bone scaffolds [5] limited by the resorption being more rapid than the new bone formation for the former and brittleness, difficulty in molding, and minimal resorption for the latter [1]. Tissue engineering techniques for bone regeneration have been developed. Mesenchymal stem cells (MSCs) can be induced to differentiate into multiple mesodermal lineages, including bone and cartilage [6]. Mesenchymal condensation, characterized by the formation of high-density cell aggregates, occurs during the early development of several tissues [7] and involves migration of the mesenchymal progenitors to the site of skeletogenesis. Culture systems that promote mesenchymal condensation are necessary to induce osteogenic differentiation in vitro, however, biochemical factors can also efficiently induce this differentiation.

Three-dimensional (3D) [8] and dynamic flow environments [9] promote osteogenic differentiation of MSCs in vitro. The 3D culture methods used for bone regeneration [8, 10] include high-cell-density cultures such as micromass cultures [11], pellet cultures [12], and 3D spheroid cultures on micropatterned substrates [10]. MSC-spheroids cultured in 3D systems are more effective than the monolayer culture systems in inducing MSC differentiation [10].

We have previously shown that spheroidal cell aggregates can be obtained by rotation culture [13]. Rotation cultures provide a 3D dynamic flow environment in vitro, which facilitates cell condensation [13, 14], enhances cell-to-cell contact, and cell aggregation [13–15], and promotes rapid and large-scale formation of spheroids [14]. We recently developed a 3D rotational cell-culture system to generate large aggregates of bone marrow (BM) stromal cells [16], using which chondrogenic differentiation was achieved without using a matrix [17, 18].

We have shown that compared to monolayer cultures, rotational cultures are more similar to in vivo cellular environments and more conducive to osteogenesis [13] and promote earlier (day 7) osteocalcin synthesis and calcium deposition.

This study was aimed at assessing the effectiveness of the MSC-spheroids generated by the rotation culture system in the repair of cranial bone defects in a well-established rat model [19] and compared the bone formation ability of MSC-spheroids and β-TCP.

## Materials and methods

### Animals

**Fig. 1:**
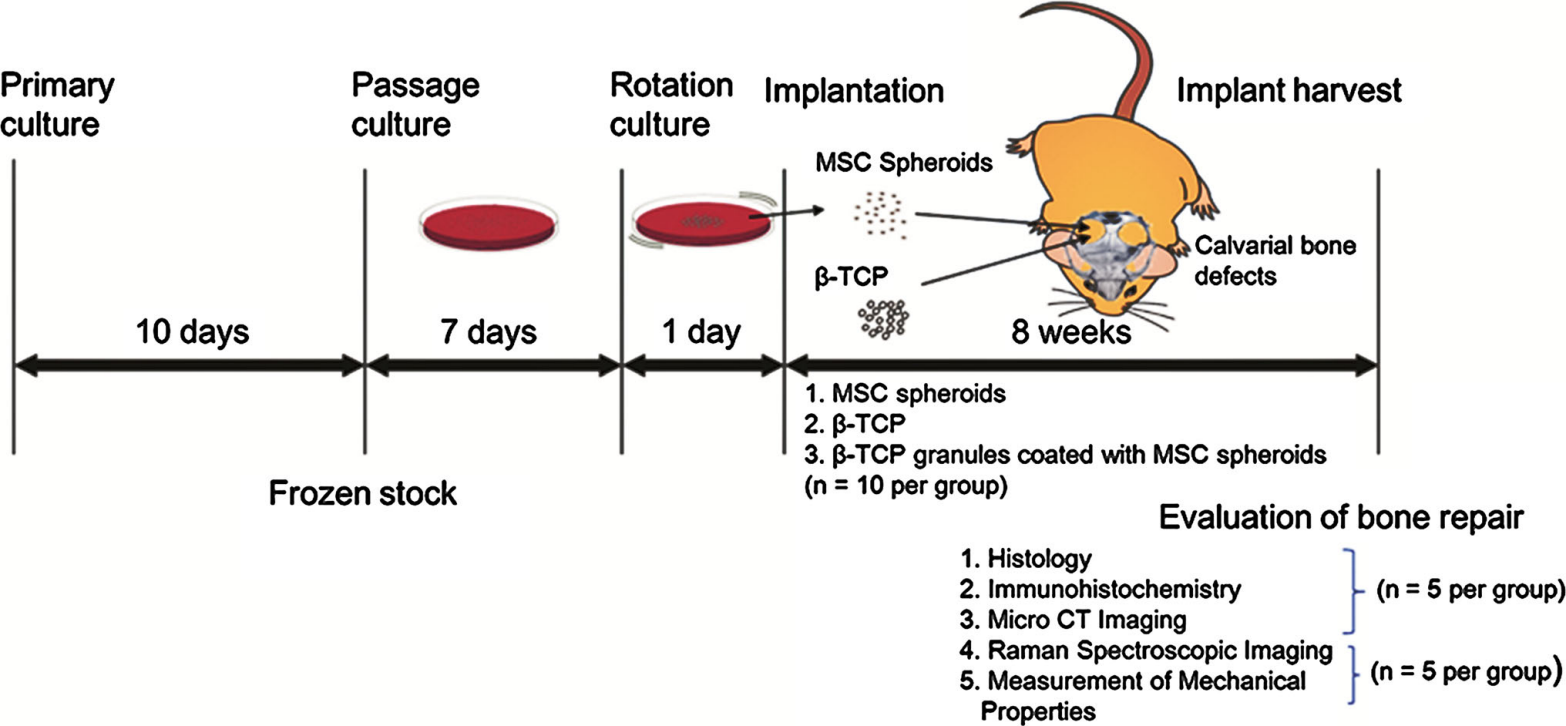
Outline of the experimental design: human mesenchymal stem cells (MSCs) were obtained, cultured in vitro for 10 days, and frozen in aliquots until further use. When required, the frozen stock was thawed and cultured for 7 days, followed by rotation culture for 1 day. The resulting MSC spheroids were collected and implanted into calvarial bone defects created in rats (n = 10). Three treatment groups with the following implants were analyzed for bone regeneration: MSC spheroids, beta-tricalcium phosphate (β-TCP), and a combination of MSC spheroids + β-TCP (n = 10 rats per treatment group). After a recovery period of 8 weeks, bone regeneration at the defect sites was evaluated

Seven-week-old male F344/Jcl rats, purchased from CLEA Japan, Inc. (Tokyo, Japan), were acclimatized for a week before the experiments. All animal experiments were conducted in accordance with the European Communities Council Directive of November 24, 1986 (86/609/EEC). The study protocol (Fig. 1) was approved by the Institutional Review Board of the Graduate School of Engineering, The University of Tokyo.

### Isolation and culturing of human MSCs

Fresh BM samples of 3–4 anonymous adult donors were obtained from AllCells (Berkeley, CA), and MSCs were isolated, as described previously [20], using Histopaque-1077 (Sigma, Saint Louis, MO). The MSCs thus obtained were cultured at a density of 2.5 × 10^3^ cells/cm^2^ in a humidified 37 °C/5 % CO_2_ incubator containing an expansion medium comprising the following: low-glucose Dulbecco’s modified Eagle’s medium (DMEM; Gibco BRL, Gaithersburg, MD) supplemented with 10 % fetal bovine serum (FBS; Bio-Whittaker, Walkersville, MD), 1 ng/mL recombinant human fibroblast growth factor (FGF-2; PeproTech EC, London, UK), 100 U/mL penicillin, 100 μg/mL streptomycin, and 0.25 μg/mL amphotericin B (Gibco Invitrogen, Grand Island, NY). The medium was completely replaced after 3 days and twice every week thereafter. MSCs adhering to the tissue culture plastic plates (Becton–Dickinson, Franklin Lakes, NJ, USA) were isolated from the culture, whereas non-adherent hematopoietic cells were discarded along with the culture medium during medium replacement. On reaching about 80 % confluence (~10 days; Fig. 1), the primary MSC cultures were harvested and frozen (−80 °C) in 8 % dimethylsulfoxide (Sigma, St Louis, MO)/10 % FBS/DMEM. Eight days before the surgical procedure, the cells were thawed and passaged twice over 7 days in the expansion medium.

### Formation of MSC-spheroids by rotation culturing

The MSC-spheroid suspension was prepared using a 3D rotational culture system, as described previously [13–17, 21]. In brief, the MSCs were detached from the tissue culture plate using 0.25 % trypsin/1 mM EDTA (Gibco Invitrogen, Grand Island, NY, USA) and resuspended at 1.0 × 10^7^ cells in 5-mL osteogenic medium for rotation culture. The osteogenic medium [22] comprised DMEM supplemented with 10 % FBS, 100 nM dexamethasone (Sigma, St. Louis, MO), 0.05 mM l-ascorbic acid-2-phosphate (Sigma), 10 mM sodium glycerophosphate (Sigma), 100 U/mL penicillin, 100 μg/mL streptomycin, and 0.25 μg/mL amphotericin B. The cell suspension (MSCs in osteogenic medium) was placed in plates with an ultra-low attachment-coated polystyrene surface (Costar, 6-well cluster plates; Corning Costar Corp, Corning, NY). The dishes were placed on a shaker (Taitec, Saitama, Japan), rotated at a constant speed of 70 rpm, and incubated in a humidified 37 °C/5 % CO_2_ incubator for 1 day. MSC-spheroids were formed within a day of the rotation culture. The spheroids were detached from the culture dishes by pipetting and examined by phase-contrast microscopy using an Olympus AX80 microscope (Olympus, Tokyo, Japan).

### Implantation of MSC-spheroids in rat calvarial bone defects

**Fig. 2:**
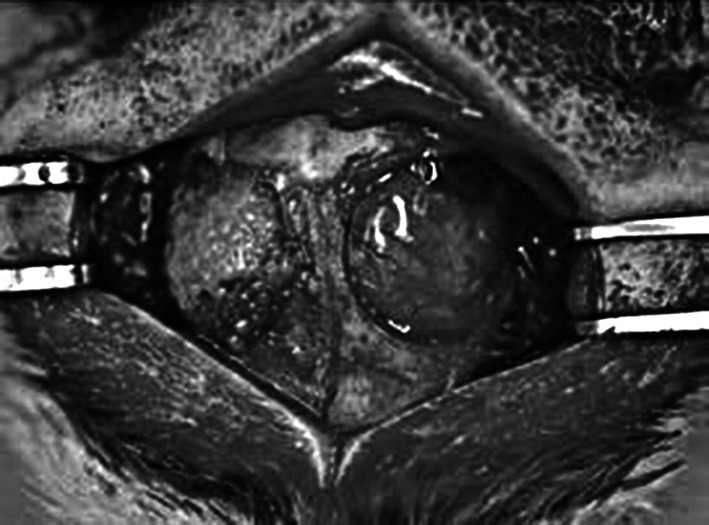
Implantation of mesenchymal stem cell (MSC) spheroids in rat calvarial defects: calvarial defects were generated bilaterally in each rat, and MSC spheroids were implanted on the *left side*, whereas the *right side* was left untreated as control

Anesthesia was induced by an intraperitoneal injection of 10 % pentobarbital (30 mg/kg body weight) (Dainippon Sumitomo Pharma, Osaka, Japan), and the surgical field was prepared with iodophor (Meiji Seika, Tokyo, Japan). Midline sagittal incisions were extended from the occipital region. With anterior and posterior subperiosteal dissection, the frontal and parietal regions of the calvaria were exposed. Bilateral cranial bone defects (diameter, 8 mm) were created (2 defects/rat) using a trephine bur (GC, Tokyo, Japan). The rats were divided into 3 equal groups, and the left-sided defects were implanted with 12.5 mm^3^ of MSC-spheroids, β-TCP granules [23] (Osferion; Olympus Terumo Biomaterials, Tokyo, Japan), or MSC-spheroids coated with β-TCP granules. The MSC-spheroid suspension volume was measured using a 1 ml syringe. The untreated right-sided defects served as the internal controls (Fig. 2). After 8 weeks, the rats were killed by cervical dislocation after pentobarbital administration (Fig. 1).

### Live/dead staining for assessment of MSC viability

To confirm their survival before surgical transplantation, MSC-spheroids were incubated with 2 μM calcein acetoxymethyl ester (calcein-AM) and 4 μM propidium iodide (Dojindo, Kumamoto, Japan), fixed in a 10 % formalin neutral buffer (Sigma, St Louis, MO) solution for 30 min, and placed in a humidified 37 °C/5 % CO_2_ incubator for 20 min. Cells were visualized using a DMIRE2 fluorescence microscope (excitation/emission, 485 nm/525 nm; Leica, Wetzlar, Germany).

### Histological assessment and immunohistochemical staining

The site of implantation and adjacent tissue were excised, fixed in 10 % phosphate-buffered formalin, and decalcified in Plank-Rychlo’s solution (MUTO Pure Chemicals Co., Tokyo, Japan) [24]. They were then fixed in 4 % paraformaldehyde (Wako, Osaka, Japan), embedded in paraffin, cut into 4-µm-thick sections, and stained with hematoxylin-eosin (HE; Sigma, St. Louis, MO). They were then incubated in 10 % goat serum (Dako, Glostrup, Denmark) for 1 h to suppress non-specific IgG binding.

Immunohistochemical staining for osteocalcin and osteopontin [25]—markers of late-stage osteoblast development—was performed using antibodies specific for humans and rat. Briefly, the tissue specimens were incubated in a 1:100-diluted anti-osteocalcin antibody solution (2 μg/mL, mouse monoclonal IgG; Abcam Inc, Cambridge, MA) or 1:100 diluted anti-osteopontin antibody solution (2 μg/mL, rabbit polyclonal IgG; COSMO BIO/LSL, Tokyo, Japan) at 37 °C for 1 h, followed by 3 washes in phosphate-buffered saline (Wako, Osaka, Japan). The specimens were further incubated in a horseradish peroxidase-conjugated secondary antibody (Envision System; Dako, Glostrup, Denmark) for 30 min at room temperature, followed by 3 phosphate-buffered saline washes. Then, 3,3′-diaminobenzidine tetrahydrochloride (Dako) was used as a substrate, and the sections were counterstained with Mayer hematoxylin (Dako) and observed under an Olympus BX43 microscope.

### Micro-CT analysis

The samples were examined with a micro-CT system (InspeXio SMX-90CT; Shimadzu, Kyoto, Japan; resolution, 105 μm; section-to-section distance, 105 μm) using an InspeXio scanner. The TRI/3D analysis software (RATOC, Tokyo, Japan) was used for 3D reconstruction of the regions of interest from the micro-CT images. For standardization, the measurements were made at equivalent sites in all samples.

### Raman spectroscopic analysis

Raman spectroscopic analysis was used to detect the subtle biochemical changes and spectral characteristics, as described previously [26]. All Raman spectra were collected from 116.0371–3440.7139 (cm^−1^) using a Nicolet Almega XR dispersive Raman spectrometer (Thermo Fisher Scientific, Waltham, MA) at laser wavelength of 785 nm and 5.00 s of accumulation per exposure, with five exposures for each recorded spectrum, and processed using the OMNIC spectroscopy software (Thermo Fisher Scientific).

### Measurement of mechanical properties

Samples of native calvarial bones (obtained from a healthy male rat of the same strain and age) and the left and right calvarial bones from the three groups were subjected to the three-point bending test on Shimadzu 5 kN Autograph (AGS-G 5 kN; Shimadzu) [27]. Samples were trimmed into circles (diameter, 8 mm) and tested at room temperature, with a 3-mm support span. Load was applied at a constant deformation rate of 0.3 mm/min, at the upper anterior midpoint of the sample until failure. Load-deformation curves were recorded and the displacement was calculated in millimeters directly from the curves. Young’s modulus and maximum bending stress are commonly used parameters to assess the stiffness of bones during elastic deformation [28]. Maximum bending stress is the maximum stress induced at a point in bones subjected to bending loads.

Young’s modulus is defined as the ratio of stress to strain within the elastic region of the stress strain curve (before the yield point). The three-point bending Young’s modulus (E) and maximum bending stress (σmax) were determined as follows:

$${\text{Young's modulus }}\left( {\text{E}} \right) \, = \, \Delta {\text{P}} \cdot {{1^{3} } \mathord{\left/ {\vphantom {{1^{3} } {\Delta {\text{y}}}}} \right. \kern-0pt} {\Delta {\text{y}}}} \cdot 4{\text{bh}}^{3}$$

$${\text{Maximum bending stress}}\,(\sigma \hbox{max} ) = {{3\Delta {\text{P}}1} \mathord{\left/ {\vphantom {{3\Delta {\text{P}}1} {2{\text{bh}}^{3} }}} \right. \kern-0pt} {2{\text{bh}}^{3} }},$$

where ΔP is the maximum force applied to the object (N); l, span of the support points (mm); Δy, amount by which the length of the object changes (mm); b, original width of the object (mm); and h, original thickness of the object (mm) [28, 29].

### Statistical analysis

Measurements for each sample were taken once. Intergroup differences in bone regeneration were evaluated by ANOVA; *P* < 0.05 was considered statistically significant. Quantitative data was expressed as mean ± standard error (SE).

## Results

### Assessment and implantation of the MSC-spheroids

**Fig. 3:**
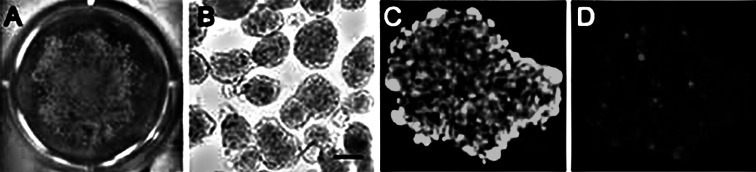
Characteristics of the mesenchymal stem cell (MSC) spheroids: **a** light microcopy image showing free-floating multicellular spheroids formed from human bone marrow-derived mesenchymal stem cells in rotation culture (1 day), without the use of any scaffold. **b** Phase-contrast microscopy of spheroids obtained from human MSCs after 1 day in rotation culture (scale, 100 μm). **c** Fluorescence microscopy of spheroids stained with calcein-AM to visualize live cells that appear *green*. **d** Fluorescence microscopy of spheroids stained with propidium iodide to visualize the nucleus (*red*) of dead cells (Color figure online)

Numerous free-floating multicellular spheroids (Fig. 3a, b) (average diameter, 100–200 μm) (Fig. 3b) were obtained using the rotation culture system. Assessment of cell viability of the spheroids before implantation was done by fluorescence microscopy after staining with calcein-AM and propidium iodide. Viable cells stained green (Fig. 3c), whereas the nuclei of dead cells stained red (Fig. 3d). Thus, a high level of viability was confirmed in the spheroids. About 3.0 × 10^7^ MSC spheroids were implanted spontaneously to the defect wall.

### Histological and immunohistochemical analysis

**Fig. 4:**
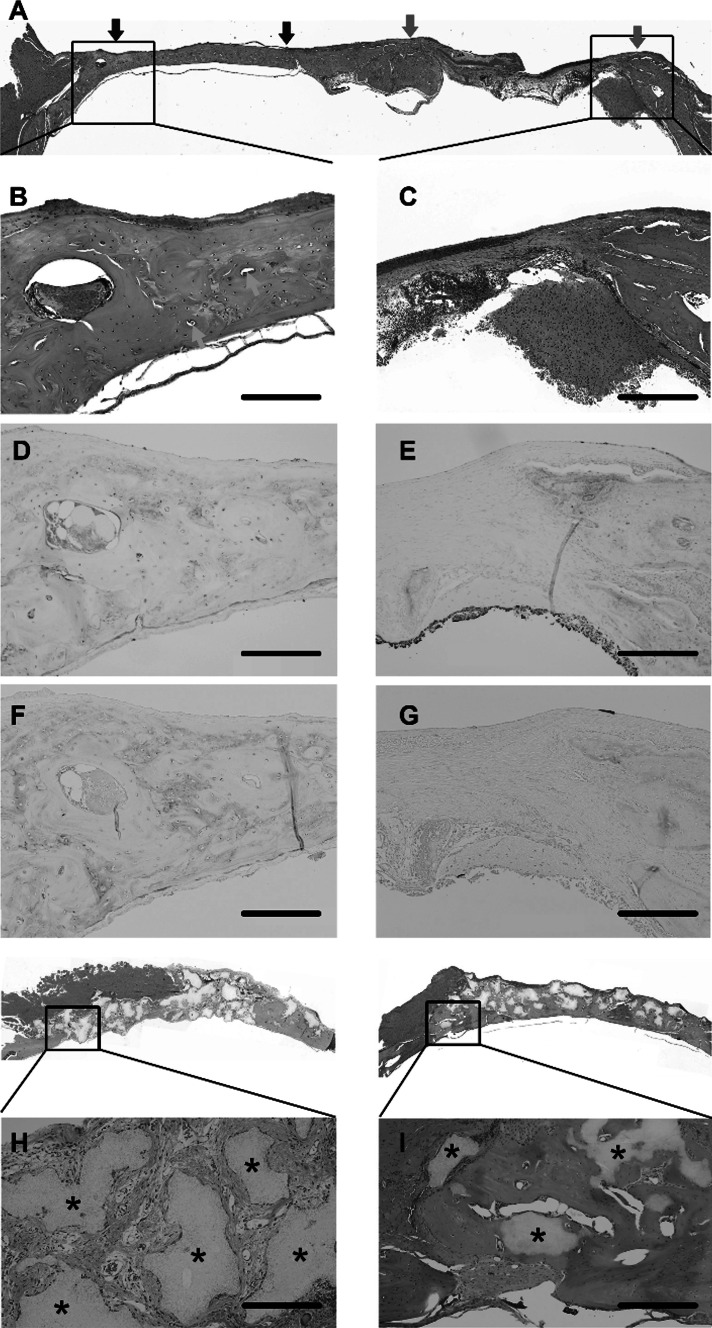
Histological assessment of bone regeneration at 8 weeks post-implantation: Bone regeneration was examined in calvarial bone defects at 8 weeks after implantation of mesenchymal stem cell (MSC) spheroids, β-TCP granules alone, and MSC spheroids + β-TCP, and was compared with the untreated defect sites. **a** Hematoxylin and eosin (HE) staining of the whole bone section showing the implanted area (*left side*) and the untreated control area (*right side*). **b**, **c** Magnified view of the HE-stained, MSC spheroid-implanted site (**b**) and control site (**c**). The control site showed only a thin band of fibrous connective tissue in the defect area along with minimal new bone formation (**c**). In contrast, at the MSC spheroid-implanted site, new vascularization was apparent, along with a significant amount of new bone and bone proteins throughout the defect area (**b**). **d**, **e** Immunohistochemical staining to visualize distribution of osteocalcin, MSC-implanted site (**d**) and untreated defect site (**e**). **f**, **g** Immunohistochemical staining to visualize distribution of osteopontin in the MSC-implanted site (**f**) and untreated defect site (**g**). **h**, **i** Defect site implanted with β-TCP granules alone (**h**) and β-TCP + MSC spheroids (**i**). The β-TCP implant site showed disintegrating tissue, fibrous tissue, and blood vessels between β-TCP granules (shown by *asterisks*). The site implanted with spheroids + β-TCP showed formation of new bone with fewer interspersed β-TCP granules (shown by asterisks). Scale, 500 μm

The defect sites (Fig. 4a) were identified visually. HE staining showed vascularization and bone regeneration in the MSC-implanted section on the left calvarial bone (Fig. 4b), but loose, fibrous granulation tissue, without bone formation, at the control sites (Fig. 4c).

Immunohistochemical staining for osteocalcin and osteopontin at postoperative week 8 showed dense staining, indicating new bone formation, at the implanted sites (Fig. 4d, f, respectively), but poor staining at the control sites (Fig. 4e, g, respectively) in the MSC-spheroid group; minimal bone formation (Fig. 4h) at the β-TCP-implanted sites; and minimal, non-uniform bone formation at the MSC-spheroids + β-TCP-implanted sites (Fig. 4i). This suggested that the β-TCP scaffold possibly restricts the bone regenerative ability of the MSCs. Additionally, β-TCP granules appeared to persist without resorption.

### Micro-CT analysis

**Fig. 5:**
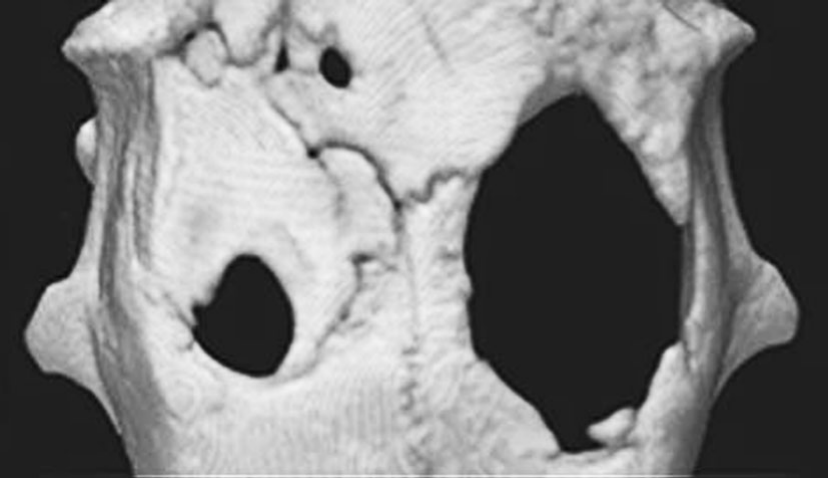
Micro-computed tomography (CT) images of a rat skull at 8 weeks after implantation: Calvarial defects created bilaterally in rats were implanted with mesenchymal stem cell (MSCs) spheroids (left side) or were left untreated (*right side*). At 8 weeks post-implantation, the defect sites were examined by micro-CT. At the implant site, the diameter of the *cylindrical holes* had narrowed, (*left side*), whereas at the control site, there was significantly less bone regeneration (*right side*) (**a**). Micro-computed tomography (CT) images of defect site implanted with β-TCP granules alone (**b**), micro-computed tomography (CT) images of defect site implanted with β-TCP granules and MSC spheroids (**c**)

Micro-CT analysis of the MSC-spheroid implants revealed new bone formation at the implanted sites, but little bone ingrowth from the defect margin without new bone formation in the central area of the control defects (Fig. 5). This may be due to limited expression of the markers osteocalcin and osteopontin at the edges of the untreated, control defect sites, but abundant expression at the MSC-spheroid-implanted sites.

### Raman spectra analysis of the bone tissue

**Fig. 6:**
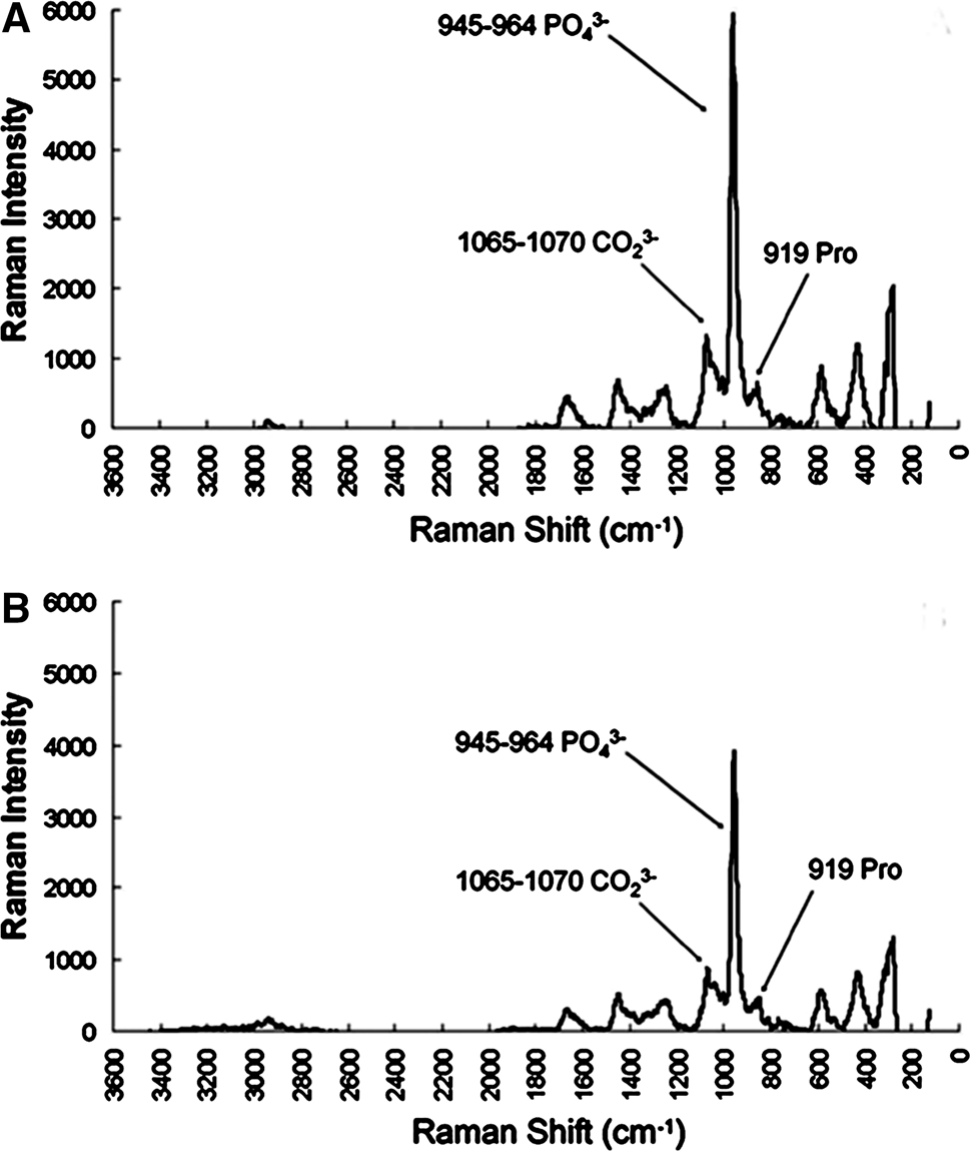
Bone tissue spectra obtained by Raman spectroscopy: **a** Raman spectrum of newly formed bone from a mesenchymal stem cell (MSC) spheroid-implanted calvarial defect site. In these spectra, the background signals have been removed. **b** A typical Raman spectrum of a native calvarial bone showing the major *peaks*

Raman spectra of the MSC-spheroid-implanted samples revealed that the only resolvable mineral factor was carbonated apatite (PO_4_^3−^, 959 cm^−1^; CO_3_^2^, 1072 cm^−1^), while the only resolvable matrix factor was a collagen-dominated protein signaled by proline at 919 cm^−1^ (Fig. 6a). The Raman spectrum of the bone-like tissue at the implant sites had peaks at 1065–1070 cm^−1^, 945–964 cm^−1^, and 919 cm^−1^, as observed for the native bone (Fig. 6b). This suggests that the partial restoration of bone thickness at postoperative week eight was associated with bone mineralization, evidenced by the accumulation of collagen proteins and CP minerals.

### Measurement of mechanical properties

**Fig. 7:**
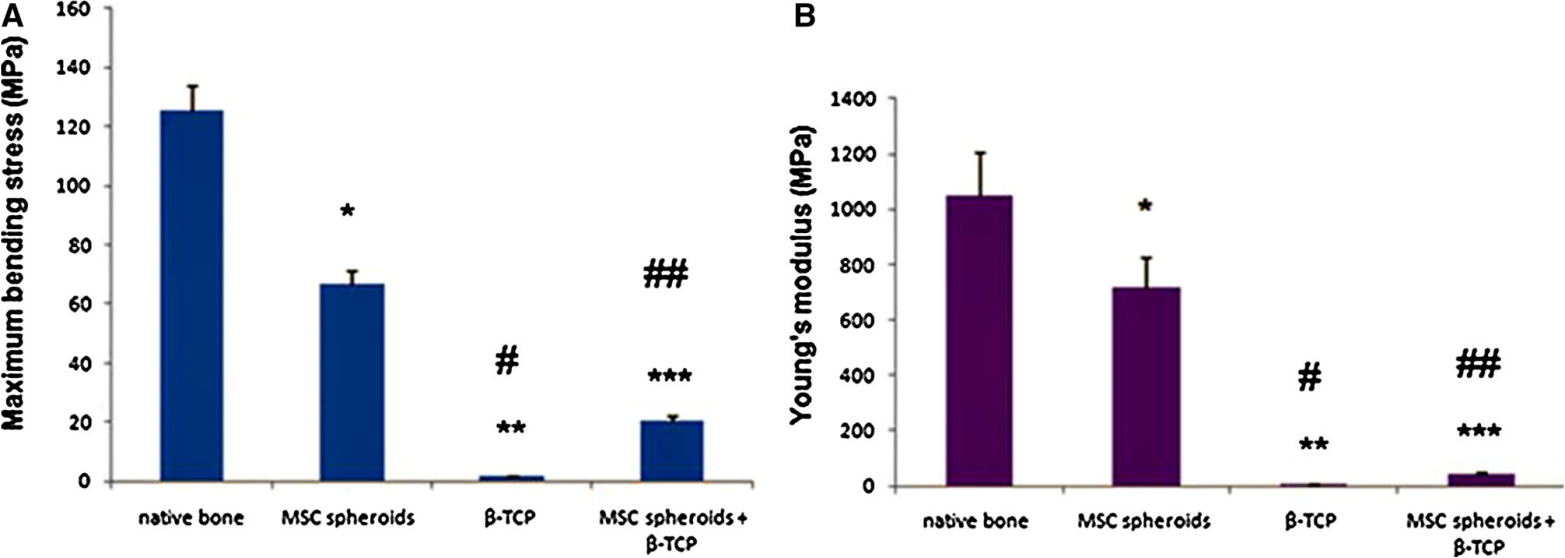
Measurement of dynamic mechanical properties of newly formed bone: Mechanical performance of bone samples obtained from the implantation sites of MSC spheroids, β-TCP, and MSC spheroids + β-TCP was compared with that of the native bone at 8 weeks post-implantation. **a** Maximum bending stress of the indicated bone samples. *Values* plotted are mean ± standard error (SE) (n = 5 in each group). *P < 0.05,**P < 0.01, ***P < 0.005 versus native bone; ^#^ *P* < 0.05, ^##^ *P* < 0.01 versus MSC spheroid group. **b** Young’s modulus for the indicated bone samples. *Values* plotted are mean ± SE (n = 5 in each group). *P < 0.05, **P < 0.01, ***P < 0.005 versus native bone; ^#^ *P* < 0.05, ^##^ *P* < 0.01 versus MSC spheroid group

Bone strength and elasticity of new bones in the MSC-spheroid group were 50 and 60 % of the native bone, respectively (*P* < 0.05) (Fig. 7a, b), and the corresponding values were 15 and 5 % in the MSC-spheroid + β-TCP group (both *P* < 0.005). Thus, the bone tissue regenerated by MSC-spheroid implantation showed partial improvement in mechanical strength and elasticity. The mechanical properties of the β-TCP group are rather weaker than those of the β-TCP + cells.

## Discussion

We investigated the effectiveness of implanting human MSC-spheroids prepared by a simple rotation culture without using a 3D matrix in the repair of critical-sized bone defects (8 mm) in the rat calvaria. Previous studies on calvarial bone regeneration have involved the use of genetically engineered MSCs [30–33], and various synthetic biodegradable 3D scaffolds for MSC implantation, including polyvinylidene chloride (PVDC) films [34], natural bone mineral (Bio-Oss) with β-TCP [35], and poly (lactide-co-glycolide) scaffolds [36] have been used. CP ceramics, including β-TCP and hydroxyapatite, are effective as scaffolds for BM-derived MSCs for bone regeneration and vascularization [37, 38], with hydroxyapatite affording greater bone volume than β-TCP. Further, a mechanically stable synthetic framework (polycaprolactone scaffold) combined with a biomimetic hydrogel (fibrin glue) can serve as a 3D matrix for the seeding of MSCs to promote osteogenesis [39]. However, the bone-regenerative potential of scaffold-free MSC-spheroids remains unexplored. This is the first study to show successful bone regeneration by MSC-spheroid implantation in calvarial bone defects without using a 3D matrix. Our rotation culture system facilitates cell condensation and rapid and large-scale formation of MSC-spheroids. Thus, spheroids were generated without using artificial materials or scaffolds, contained only cells and a self-secreted matrix; therefore, they could be obtained as a suspension, which adhered well to the underlying bone during the filling of the calvarial defect facilitating recovery.

Spheroid size is an important factor affecting cell functions, such as extracellular matrix formation and viability. The optimal diameter of the MSC-spheroid microdomains formed by chondrocytes is 200–600 μm [14]. Spheroids obtained in this study had a diameter of 100–200 μm, which approximates the minimal distance for material exchange by diffusion in a living body [40], and presumably facilitates the survival of MSC-spheroids in vivo. Testing for cell viability before implantation confirmed that practically all cells within the spheroids were viable. Future studies are required to assess MSC viability at post-transplantation week eight. Time-series experiments such as reporter assays may help reveal the exact in vivo mechanism of MSC-mediated new bone formation.

Histological examination for osteocalcin and osteopontin performed at post-implantation week 8 revealed the limited expression of these markers at the edges of the untreated, control defect sites, but abundant expression at the MSC-spheroid-implanted sites; minimal expression was noted at the MSC-spheroid + β-TCP-implanted sites. OPN/OCN expression reveal the differentiation of human MSCs into osteoblasts.

Because histological analysis of tissue slices may not provide information about the entire sample volume, we also performed micro-CT analysis. This enabled the examination of consecutive sections of the same region in the specimens and showed a striking difference in the extent of repair at the implanted and control sites.

Raman spectroscopy is the most specific test to identify the chemical composition of a given material. Unlike the case with the β-TCP and MSC-spheroids + β-TCP groups (data not shown), the Raman spectral characteristics of the new bones formed by the MSC-spheroid implantation were similar to those of native bones, indicating bone mineralization.

Bones formed by MSC-spheroid implantation had nearly 50 % of the strength healthy/native bone at post-implantation week eight. Bone strength in the MSC-spheroid + β-TCP group was 15 % that of the native bone, which suggests that β-TCP possibly hinders healing by reducing MSC cell mobility and cell migration from the implant to the bone surface. Further, β-TCP granules persisted at the interfaces between the granules and newly formed bone tissue, preventing uniform bone formation. Although the exact reason for this remains unclear, it may be explained by host-related biological factors and particle size of β-TCP granules. Particle release from bioceramic scaffolds can impair the ability of BM-derived human MSCs to proliferate or mature into functional osteoblasts [41]. Further, the β-TCP granules used in our study were large (0.5–1.5 mm); smaller bioceramic particles (<20 µm) elicit a stronger inflammatory than larger particles (40–80 µm and 80–200 µm) and facilitate osteogenesis [42, 43].

Donor MSC-spheroids may simply induce an osteogenic response, enhancing osteoblast recruitment to the implantation site, rather than acting as sources of new bone.

## Conclusion

In conclusion, osteoblastic differentiation of human BM MSCs can be induced by a rotation culture and the bone formed after MSC-spheroid implantation has histological and spectral characteristics similar to native bone and good mechanical strength. Thus, MSC-spheroids obtained by a simple rotation culture should be considered a new approach in the reconstruction of large calvarial bone defects without using synthetic scaffolds.
